# Advances in Polymer Binder Materials for Lithium-Ion Battery Electrodes and Separators

**DOI:** 10.3390/polym15234477

**Published:** 2023-11-21

**Authors:** Siyeon Lee, Heejin Koo, Hong Suk Kang, Keun-Hwan Oh, Kwan Woo Nam

**Affiliations:** 1 Graduate Program in System Health Science and Engineering, Department of Chemical Engineering and Materials Science, Ewha Womans University, Seoul 03760, Republic of Korea; 2 Program in Environmental and Polymer Engineering, Department of Polymer Science and Engineering, Inha University, Incheon 22212, Republic of Korea; 3 Hydrogen Energy Research Center, Korea Research Institute of Chemical Technology (KRICT), Daejeon 34114, Republic of Korea

**Keywords:** lithium-ion battery binder, conventional binders, next-generation binders, polymer

## Abstract

Lithium-ion batteries (LIBs) have become indispensable energy-storage devices for various applications, ranging from portable electronics to electric vehicles and renewable energy systems. The performance and reliability of LIBs depend on several key components, including the electrodes, separators, and electrolytes. Among these, the choice of binder materials for the electrodes plays a critical role in determining the overall performance and durability of LIBs. This review introduces polymer binders that have been traditionally used in the cathode, anode, and separator materials of LIBs. Furthermore, it explores the problems identified in traditional polymer binders and examines the research trends in next-generation polymer binder materials for lithium-ion batteries as alternatives. To date, the widespread use of N-methyl-2-pyrrolidone (NMP) as a solvent in lithium battery electrode production has been a standard practice. However, recent concerns regarding its high toxicity have prompted increased environmental scrutiny and the imposition of strict chemical regulations. As a result, there is a growing urgency to explore alternatives that are both environmentally benign and safer for use in battery manufacturing. This pressing need is further underscored by the rising demand for diverse binder research within the lithium battery industry. In light of the current emphasis on sustainability and environmental responsibility, it is imperative to investigate a range of binder options that can align with the evolving landscape of green and eco-conscious battery production. In this review paper, we introduce various binder options that can align with the evolving landscape of environmentally friendly and sustainable battery production, considering the current emphasis on battery performance enhancement and environmental responsibility.

## 1. Introduction

LIBs have gained widespread use in various applications due to their impressive characteristics such as high energy density, efficient charging/discharging, and minimal self-discharge. Over the last decade, there has been a noticeable shift toward the development of advanced lithium-ion batteries (LIBs) with increased charge capacity and power density. These improved batteries are designed for use in electric vehicles (EVs), hybrid electric vehicles (HEVs), aerospace applications, and autonomous electric devices like hybrid solar batteries [1]. In contrast to consumer electronics, the automotive sector imposes stricter technical demands, including longer calendar life (10 years), a greater number of charge–discharge cycles (1000 cycles), a wider operating temperature range (−30 to 52 °C), and a lower cost target (USD 100 per kWh). These heightened performance criteria account for the significant 17-year gap between the introduction of LIBs in consumer products compared to their adoption in automotive applications [2].

Binders play a crucial role in lithium-based rechargeable batteries by preserving the structural integrity of electrodes. Despite their small percentage in the overall electrode composition, binders have a significant impact on battery performance [3]. In order to achieve reliable and consistent cycling performance in the electrode structure, it is necessary to appropriately adjust and customize binders and additives [4]. Polyvinylidene fluoride (PVDF) binder is a popular choice because of its electrochemical stability and its capacity to flexibly handle mechanical compression during the charging and discharging processes. However, conventional polymer binders such as PVDF have disadvantages such as poor mechanical properties and thermal stability.

For anodes, researchers have experimented with modified binders to overcome limitations associated with conventional binders. They have introduced binders such as a gradient hydrogen-bonding binder and self-healing poly(ether-thioureas) (SHPET) polymer binder to enhance the performance of silicon(Si)-based anodes [5,6]. In the context of cathodes, PVDF binder is commonly used. Researchers are working on next-generation polymer binders to stabilize cathode materials like layered LiCoO_2_ (LCO) at high voltages. These binders include dextran sulfate lithium (DSL), S-binders, and other innovative materials like fluorinated polyimide (PI-FTD) and poly(imide-siloxane) (PIS). Researchers are working on enhancing the thermal stability and electrolyte wettability of polyolefin separators through surface coatings and unique binder materials. In summary, researchers are actively exploring various binder materials and approaches to enhance the performance, safety, and environmental aspects of LIBs, including both anode and cathode binders as well as separator binders.

In most cases, binders have a significantly low mass ratio because they do not participate in the battery’s electrochemical reactions. Their primary role is to integrate the various components of the electrode into a cohesive entity and uphold the physical structure of the electrode. This is crucial for facilitating efficient electron and ion movement. Consequently, even though they are typically used in small quantities, binders have a significant impact on the safety and electrochemical performance of LIBs [3]. This review paper provides a comprehensive examination of binders in LIBs. It covers an overview of binders used within the cathode, anode, and separator, both those in current commercial use and newly improved ones. This paper aims to enhance the understanding of binder types and characteristics and their influence on battery technology. Furthermore, it is expected to serve as important guidance for future developments in lithium-ion battery technology.

The widespread utilization of N-methyl-2-pyrrolidone (NMP) as a solvent in electrode processing for lithium batteries has long been a standard practice. However, recent concerns over its high toxicity have triggered heightened environmental scrutiny, resulting in the imposition of stringent chemical regulations. Consequently, there is a growing imperative to seek alternatives that are both environmentally benign and safer for use in battery production. This pressing need is underscored by the demand for diverse binder research within the lithium battery industry. With the current emphasis on sustainability and environmental responsibility, it is imperative to explore a range of binder options that can accommodate the evolving landscape of green and eco-conscious battery manufacturing. In response to these challenges, Zhou, H. et al. investigate dihydrolevoglucosenone (Cyrene), a relatively new entrant among green dipolar aprotic solvents. Cyrene shares solvent properties with NMP, making it an attractive candidate for substitution. Their research explores the feasibility of employing Cyrene as a replacement for NMP in the fabrication of NMC 811 cathodes, with a particular focus on its suitability as a binder solvent and its impact on battery performance. While Cyrene initially exhibits limitations in solubilizing PVDF binder at room temperature, their study demonstrates that temperature adjustments can significantly enhance its solubility. High-temperature electrode processing with Cyrene exceeding 80 °C yields promising results comparable to traditional NMP-based electrode manufacturing. These findings not only substantiate Cyrene’s potential as a greener alternative to NMP but also underscore the need for continued exploration of diverse binder options to align with the evolving principles of sustainability and environmental stewardship in the lithium battery industry [7].

## 2. Anodes

### 2.1. Conventional Binders for LIB Anodes

#### 2.1.1. Graphite Anode Binders

**Figure 1:**
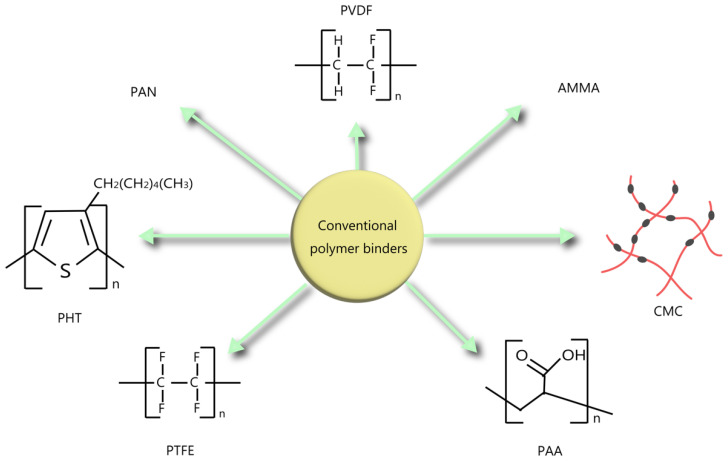
Schematic representation of conventional polymer binders for LIB anodes.

For over two decades, graphite has been extensively utilized as an anode material due to its favorable characteristics, including high conductivity, low cost, and good capacity retention. However, the performance of graphite electrodes is significantly influenced by various factors such as conductive materials and binders present in the electrode composition [4]. In traditional first-generation LIBs, graphite is commonly employed as the anode material. Nevertheless, the inclusion of a conductive binder may result in suboptimal packing density of the electrode, thereby restricting the volumetric energy density [8]. Figure 1 illustrates several structural formulas and graphical representations of various types of traditional polymer binders used in LIBs.

PVDF is frequently chosen as the preferred binder for graphite anodes and Li_1.05_Ni_0.33_Mn_0.33_Co_0.33_O_2_(NCM)-based electrodes due to its electrochemical stability and flexibility, allowing for mechanical compression and decompression during the charging and discharging cycles [9]. The reactivity of PVDF with lithiated graphite and metallic lithium has raised significant concerns regarding the thermal runaway of LIBs under abusive conditions. This is due to the observation that the reaction between PVDF and metallic lithium can result in a high enthalpy because metallic lithium is often present in LIBs during over-discharging or charging processes at low temperatures. Therefore, it is deemed necessary to explore alternative binders that can overcome the limitations of PVDF binders. Zhang et al. conducted DSC investigations on positively charged anodes and reported that an exothermic reaction, commencing at approximately 230 °C and reaching its maximum at around 300 °C, was likely attributed to the interaction between the PVDF binder and metallic lithium [10]. During the 1980s, PTFE (polytetrafluoroethylene) resin found extensive use as a binding agent for both anode components within LIBs due to its exceptional resistance to chemicals and heat, as well as its strong binding attributes. PTFE’s aqueous dispersion exhibited characteristics of fibrillation, which greatly enhanced its ability to bind with electrode materials effectively. However, an excessive degree of fibrillation posed challenges by impeding the formation of a uniform dispersion, leading to poor connectivity with the electrode materials [11]. Polyacrylonitrile (PAN) has been widely used as a host polymer for gel polymer electrolytes (GPEs) owing to its high polarity, excellent electrochemical stability, and solubility in non-aqueous liquid electrolytes [12]. However, PAN could not properly fulfill the role of a binder for electrodes due to its high crystallinity [12]. It was revealed that electrode films bonded with PAN were too fragile to adhere to the metal current collectors (copper for graphite anodes and aluminum for the cathode). Hence, it was crucial to chemically modify PAN to enable its function as a binder. To address the limitations of both PAN and PVDF, Zhang and Jow selected a copolymer called poly(acrylonitrile-methyl methacrylate) (AMMA) with a composition ratio of 94% acrylonitrile (AN) and 6% methyl methacrylate (MMA) [12]. Their research concluded that AMMA is a suitable binder for both graphite anodes and lithium transitional metal oxide cathodes in LIBs. In detail, for graphite anodes, AMMA outperforms PVDF, which is commonly used in the current Li-ion technology. The use of AMMA binder promotes the formation of more stable solid electrolyte interface (SEI) films on the graphite anodes. In addition, the use of AMMA binder reduces the self delithiation of lithiated graphite, thus extending the calendar life of LIBs [12]. Susumu Kuwabata et al. conducted a study where they investigated the use of poly(3-n-hexylthiophene) (PHT) as a binder for graphite anodes in LIBs. The electrochemical properties of a composite film composed of synthetic graphite and PHT were examined [13]. The graphite/PHT film exhibited a charge capacity of 43.5 mAh g^−1^ and a coulombic efficiency of 94.6% within a voltage range of 2.0 to 0 V. It demonstrated a specific capacity of 312 mAh g^−1^ for the deintercalation of Li^+^ ions, with a graphite utilization rate of 0.92, comparable to the graphite/PVDF electrodes. The use of PHT as a binder helped reduce the irreversible capacity during the initial cycle, and the electrically conductive properties of n-doped PHT facilitated binding with graphite without mechanical pressing [13].

#### 2.1.2. Si-Based Anode Binders

Si-based anodes for LIB have gained considerable attention due to their much higher specific capacity than conventional graphite. However, the high-capacity Si particles undergo significant volume changes during the insertion and extraction of Li^+^ ions. These volume fluctuations make it difficult to select an appropriate binder that can effectively bind the active materials in the anode. The conventional binder, PVDF, forms weak van der Waals forces with the Si particles, which are unable to accommodate the large changes in particle spacing. Consequently, PVDF becomes inefficient at maintaining particle cohesion and electrical conductivity within the anode, which are essential for battery performance. To achieve stable high-capacity anodes, it is crucial to develop more efficient binders [12]. There is still a need to explore the development of matrixes that can effectively handle the volume expansion of Si while remaining commercially viable. For Si electrodes, binders can play a critical role in creating a soft matrix that provides the necessary mechanical and electrochemical stability as well as electronic conductivity [14]. In their research, Shen, Lanyao et al. present the use of in situ thermally cross-linked PAN as a binder for Si-based anodes in LIBs [15]. They demonstrate that the electrode offers impressive life cycle and rate performance, retaining a reversible capacity close to 1450 mAh g^−1^, even after 100 cycles. The enhanced electrochemical functionality of these Si electrodes results from the generation of cross-linked structures and conjugated PAN induced by heat treatment. This work paves the way for further investigation into other polymers that could be used for both anode and cathode electrodes of rechargeable batteries [15]. Most of the research on Si anodes has primarily utilized carboxymethyl cellulose (CMC) and PVDF binders. However, a groundbreaking study by Magasinski et al. demonstrates that pure poly(acrylic acid) (PAA), which possesses mechanical properties similar to CMC but contains a higher concentration of carboxylic functional groups, can potentially outperform these conventional binders when used in Si anodes [16].

PAA-based binders have been extensively utilized in high-capacity Si anodes for LIBs. In their study, Hu et al. aimed to enhance the cycling performance of Si/graphite composite anodes (containing 15 wt% Si) by systematically investigating various PAA binders. They verified the molecular weight of these binders and established a correlation between molecular weight and cycling performance of the fabricated anodes [17]. Table 1, Table 2 and Table 3 provide data on the properties and electrochemical performance of the most commonly used anode binders, including PVDF, PAA, and CMC. Table 1 introduces the density of each polymer. Table 2 presents the electrochemical performance of an anode electrode with a silicon content of 70%. Additionally, Table 3 compares the electrochemical performances of Si electrodes using each binder. This information allows us to gain insights into the impact of each binder on the performance of the battery.

#### 2.1.3. Other Types of Anode Binders

Lithium titanium oxide (LTO) is regarded as a favorable anode material for electric vehicle usage among various LIB anodes. This is primarily attributed to its enhanced safety characteristics, resulting from its modest operating voltage and compatibility with conventional electrolytes. In contrast to graphite anodes, which operate at 1.55 V vs. Li^+^/Li, LTO operates at this lower voltage, effectively preventing the growth of lithium dendrites and the excessive formation of SEI on the electrode surface caused by electrode–electrolyte breakdown. Consequently, LTO significantly enhances the safety of the battery system [22].

Although PVDF is widely recognized as the most common and traditional binder, valued for its chemical and electrochemical stability, it has drawbacks, including toxicity and the need for expensive organic solvents for dissolution. Consequently, PVDF is less favored for large-scale and stringent cell chemistry applications. Additionally, PVDF-based electrodes require elevated temperatures for drying. Conversely, there is growing interest in water-based binders compared to conventional non-aqueous ones. Water-based binders offer advantages such as cost-effectiveness, low toxicity, electrochemical stability, compactness, enhanced electrolyte wettability, increased active material ratio, rapid drying options, and simplified control requirements in electrode formulation, making them more appealing [22].

Recently, efforts have been made to enhance the electrochemical performance of LTO using CMC as a water-based binder. LTO anodes with CMC binders exhibit superior electrochemical performance when compared to conventional PVDF binders, owing to improvements in electrode kinetics, reduced charge transfer resistance, lower apparent activation energy, and decreased apparent diffusion activation energy.

In line with this direction, Karuppiah et al. have introduced a new aqueous binder from the acrylic family, known as LA132 (-[-R1-R2-CH2-CH(CN)]n-), characterized by low charge transfer resistance and moderate adhesive properties due to the strong polar CN group in its main chain segment. This binder is designed for use with LTO anodes [22].

Wang, R. et al. have examined the electrochemical characteristics of CuO electrodes when employing different binders. Additionally, they have explored the adhesive qualities of organic PVDF binders and aqueous binders such as SBR + CMC and LA133, which can be adjusted based on the weight ratio of the conductive slurry. Their results indicate that when PVDF is used as the binder, there is a risk of the active material detaching from the current collector. In contrast, SBR + CMC and LA133 demonstrate superior bonding performance. Notably, electrodes prepared with an 80:10:10 slurry ratio of SBR + CMC binder exhibit exceptional electrical conductivity, efficient charge transfer, strong binding capability, impressive cycling performance, and favorable rate performance. This ultimately leads to outstanding electrochemical performance. Consequently, their research establishes the feasibility of manufacturing anode materials for lithium-ion batteries using cost-effective aqueous SBR + CMC binders instead of toxic solvents like NMP and expensive PVDF. As a result, this approach can enhance the electrochemical properties of batteries, reduce costs, and contribute to environmental preservation [23].

### 2.2. Next-Generation Anode Binders

**Figure 2:**
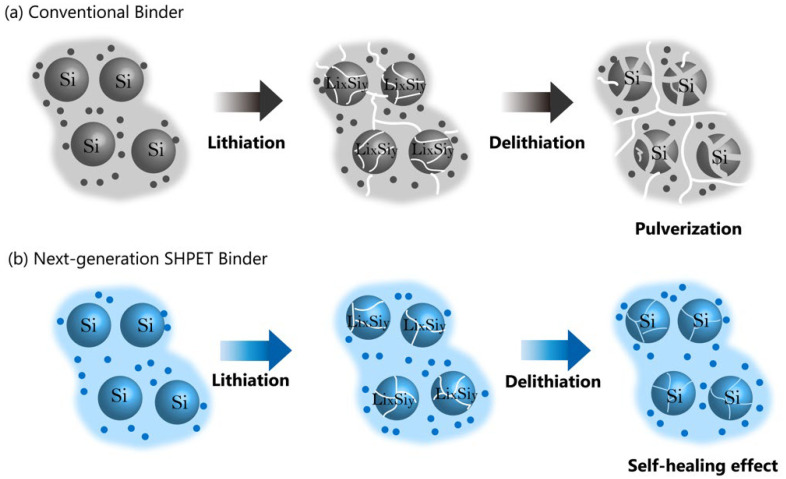
Schematic representations of lithiation/delithiation of Si particles using (**a**) conventional binder and (**b**) the SHPET binder.

Hu et al. have launched a gradient hydrogen-bonding binder for Si-based anodes with high capacity, holding great potential for next-generation LIBs [5]. The well-defined gradient hydrogen bonds in the binder play a crucial role in alleviating the substantial stress experienced by the Si anode, as they can sequentially cleave and dissipate energy. This unique feature helps prevent sudden failure of the binder structure commonly observed in conventional binders that lack gradient energy dissipation [5]. In order to tackle the challenge posed by the large volume changes in Si particles during charging and discharging cycles, researchers developed a self-healing binder capable of repairing damage to Si anodes in real time [6]. Chen et al. synthesized a novel self-healing poly(ether-thioureas) (SHPET) polymer that exhibits a balanced combination of rigidity and softness specifically tailored for Si anodes [6]. To create the SHPET binder, a copolymerization reaction was employed. In brief, 1,2-bis(2-aminoethoxy)ethane and 1,1′-thiocarbonyldiimidazole were combined in dimethylformamide (DMF) solvent. After stirring at room temperature for 24 h, a yellow solution was produced. Subsequently, chloroform was introduced to the solution, and this diluted mixture was poured into diethyl ether. This led to the formation of a precipitate, which was collected after 20 min of centrifugation at 4000 revolutions per minute. The collected precipitate was then dissolved in chloroform once more. After this process of dissolution and precipitation was repeated twice, the final precipitate was dried in an oven at 140 °C for 12 h, resulting in the acquisition of the pure SHPET polymer [6]. Subsequently, various methods were employed to characterize the SHPET polymer. In Fourier transform infrared (FTIR) spectroscopy, two broad vibrational bands at approximately 2865 and 3283 cm^−1^ were observed, corresponding to the N-H stretching and N-H deformation vibrations, respectively. The splitting of the N-H bond vibration indicates the coexistence of trans/trans and cis/trans conformations in the SHPET polymer, resulting in non-linear and less-ordered hydrogen-bonded structures. To assess the thermal and electrochemical stability of SHPET, thermogravimetric analysis (TGA) and cyclic voltammetry (CV) tests were conducted. TGA analysis revealed that the SHPET polymer remains thermally stable within a temperature range of 25 to 270  °C. This exceptional thermal stability makes SHPET suitable as a binder for batteries operating in high-temperature conditions. The electrochemical stability of SHPET polymer was evaluated through CV cycles within a voltage range of 0.001–2  V at a scan rate of 0.5  mV/s. During the initial discharge process, a broad reduction peak appeared at around 1.0  V, indicating the decomposition of the electrolyte and the formation of the SEI. However, this reduction peak disappeared in subsequent cycles, suggesting that the pure SHPET binder contributes negligibly to the capacity of the silicon anode. Moreover, the SHPET polymer displayed remarkable chemical stability when exposed to organic liquid electrolytes, as it remained insoluble in the electrolyte solution, specifically 1 M LiPF_6_ in EC/DEC/FEC. Additionally, the SHPET binder exhibited a minimal swelling ratio of 5.75% in the liquid electrolyte, confirming its structural stability in this environment [6]. The incorporation of the self-healing binder resulted in excellent structural stability and superior electrochemical performance of the Si anode. Notably, the anode achieved a high discharge capacity of 3744 mAh g^−1^ at a current density of 420 mA g^−1^, demonstrating a stable cycle life with a capacity retention of 85.6% after 250 cycles, even at a high current rate of 4200 mA g^−1^. These remarkable results indicate that the SHPET binder facilitates rapid self healing, effectively mitigates the significant volume changes experienced by the Si anode, and successfully overcomes mechanical strain during the charging and discharging processes. Consequently, these advances have the potential to accelerate the commercialization of Si anodes in practical applications [6]. Figure 2 provides schematic representations of the lithiation/delithiation processes of SHPET binder, one of the next-generation anode binders, and compares them to schematic representations of the lithiation/delithiation processes of conventional binders to illustrate the differences. During charge–discharge cycles, the volume of the Si anode increases significantly by up to 300%, which can make the electrode structure unstable and significantly reduce capacity. To resolve this issue, commercial PAA is used to create a water-soluble polymeric binder (PAA-B-HPR), which is cross-linked by hydroxypropyl polyrotaxane (HPR) via reversible boronic ester bonds for the construction of the Si anode in LIBs [24]. When undesirable volume changes occur during lithiation and delithiation, the mobile α-cyclodextrins of the modified polyrotaxane can adjust its position, allowing the system to evenly distribute the accumulated internal stress. Furthermore, the reversible boronic ester bonds help restore any damage inflicted during the production and operation phases, thereby preserving the integrity of the electrode. Consequently, LIBs fabricated with Si anodes using PAA-B-HPR binder indicate excellent specific capacity and cycling stability in the temperature range of 25 to 55 °C. Notably, the Si@PAA-B-HPR anode exhibits a specific discharge capacity of 1056 mAh g^−1^ at 1.4 A g^−1^ following 500 cycles at an elevated temperature of 55 °C, with a mere capacity decay rate of 0.10% per cycle. This investigation paves the way for the practical use of Si anodes in LIBs [24].

## 3. Cathodes

### 3.1. Conventional Binders for LIB Cathodes

**Figure 3:**
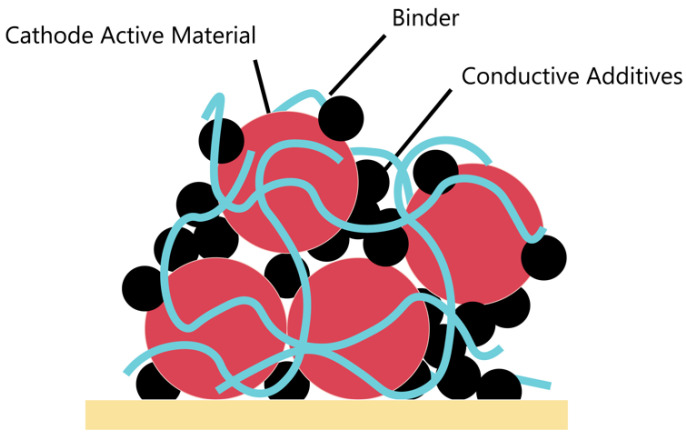
Schematic of cathode structure.

The role of the binder in the cathode is crucial for effectively binding the active material and conductive additive agent to the current collector [25]. Various types of binders have been reported, with PVDF being the dominant binder in the LIB industry [26,27]. Figure 3 demonstrates the fundamental cathode structure, and the binder helps to uniformly coat active materials and conductive additives. Table 4 provides data on the electrochemical performance of the cathodes fabricated by PVDF binders. A cathode composed of Li(Li_0.17_Ni_0.25_Mn_0.58_)O_2_ powder (80%), acetylene black (AB) (10%) as the conductive material, and PVDF binder demonstrated a discharge capacity of 238 mAh g^−1^ after 100 cycles. Furthermore, the discharge capacity after 50 cycles at a rate of 6C was 186 mAh g^−1^ [28]. Spreafico, M.A. et al. developed an industrial NMP-free process using PVDF polymer as a binder and water-based slurry. LiCoO_2_ used as a cathode material was manufactured with copper electroless coating, and a uniform coated layer was formed via a copper ion reduction process that occurs during a deposition process. In addition, it was confirmed that the PVDF binder exhibited similar performance to NMP-dispersed PVDF only when the active material was coated with copper oxide. This coating technique replaced toxic NMP with water to ensure safety and suggested the possibility of scale-up through a semi-industrial plating treatment [29]. Zheng, H. et al. analyzed the physical and electrochemical characteristics of the cathode in accordance with the ratio of PVDF as a polymer binder and AB as a conductive material. As a result, when the PVDF ratio was high, the electronic conductivity increased as the inactive material content increased due to the limited electronic conductivity of PVDF/AB. The cathode exhibits the highest cell performance at a PVDF/AB ratio of 5:4, and the electronic conductivity starts to decrease again from the ratio of 5:5, which is believed to arise from poor connectivity between particles due to lack of a binder [30].

### 3.2. Next-Generation Cathode Binders

**Figure 4:**
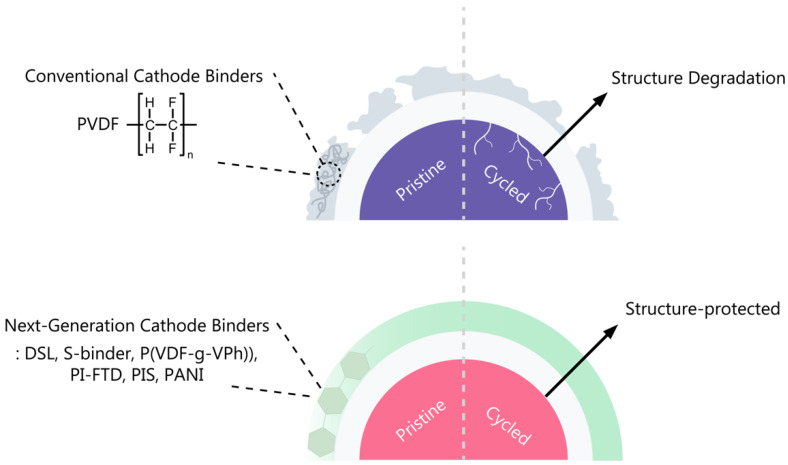
Limitations of current binders and improvements in next-generation cathode binders.

PVDF binders have certain drawbacks, including insufficient binding force and stability, expensive and environmentally harmful manufacturing processes, and limited electrical and ionic conductivity [31]. Hence, a range of polymeric binders are under investigation to address PVDF-related issues. As shown in Figure 4, structure degradation in a PVDF binder is a consequence of its low adhesive force, prompting ongoing research into diverse polymer binders capable of stabilizing cathode structures.

#### 3.2.1. LiCoO_2_

LCO was discovered by Goodenough’s group in the 1980s and remains one of the best cathode materials owing to its ability to operate at a high voltage of ~4 V [32]. Despite the high ionic conductivity of Li^+^ ions and high electrical conductivity, LCOs exhibit structural defects when charged beyond 50%, leading to the release of oxygen from the crystal lattice. These limitations restrict commercial LCOs from operating at lower voltages for long periods of time, resulting in a practical capacity of ~140 mAh g^−1^ [33].

Researchers are actively working to stabilize LCO under high-voltage conditions, and Huang H et al. introduced a new approach by replacing PVDF binders with DSL [31]. As a result of conducting FTIR within the range of 900–3600 cm^−1^ to analyze the structural differences between dextran and DSL, DSL exhibits a noticeable characteristic peak near 1200. This demonstrates the presence of sulfate groups in DSL. Furthermore, following an examination of the surface microstructure of PVDF-LCO and DSL-LCO using high-resolution transmission electron microscopy (HRTEM), relatively minor surface erosion and structural collapse were observed in DSL-LCO particles as a uniform DSL coating layer, in contrast to the serious deterioration of the surface structure of PVDF-LCO particles. DSL significantly improves the electrochemical performance of LCO by preventing irreversible bond breakage on the surface, resulting in a high reversible capacity (>200 mAh g^−1^) and a 93.4% capacity retention of its initial capacity after 100 cycles. An, J. et al. developed a Si-based binder (S-binder) that can compensate for the low adhesion and mechanical ductility of PVDF [34]. The S-binder demonstrates stronger adhesive force to the LCO surface compared to PVDF and PAA, allowing for uniform coating without self-aggregation. The LCO electrode using S-binder (Sb-LCO) exhibited excellent capacity retention of approximately 92.0% after 100 cycles at 0.33C, and Coulomb efficiency was maintained above 99%. Simulations of slot-die coating, conducted to evaluate the practical application of S-binder in the LIB industry, confirmed an improved surface tension and increased contact angle compared to PVDF-based slurries, indicating reduced fluid deformation and the production of flat-surface electrodes.

#### 3.2.2. LiNi_1−x_M_x_O_2_

NCM is attracting attention as a potential cathode material to replace LCO because of its affordability, high capacity, and excellent thermal stability [35]. The capacity and thermal stability of NCM are greatly influenced by the nickel content, with higher nickel content increasing the reversible capacity. Among NCMs, NCM-811 (LiNi_0.8_Co_0.1_Mn_0.1_O_2_) is the most commercially used variant, with a reversible specific capacity of approximately 200 mAh g^−1^ at a high voltage of 4.3 V [36]. However, cathodes with high nickel content are susceptible to moisture, have poor cycling stability, and lack thermal stability [37].

Liu, Z. et al. developed a vinylphenol-grafted PVDF binder (P(VDF-g-VPh)) that can remove reactive oxygen species through dopamine containing a phenol group [38]. This unique binder forms a thinner and more uniform cathode–electrolyte interface (CEI) layer, improving battery performance. Half cells based on NCM622 and NCM811 using P(VDF-g-VPh) binder showed an operating voltage range of 3.0–4.5 V and a capacity retention of 80.5% of the initial capacity after 200 cycles. Polyimide (PI) is a widely studied material as a battery material due to its excellent mechanical strength, high thermal stability, and inertness [39]. Pham, H. Q. et al. introduced a functional non-aqueous PI-FTD binder that exhibits high oxidation stability and thermal stability under severe conditions [40]. When applied to NCM811 cathodes, the PI-FTD binder allows for an expanded electrochemical voltage window (4.4 V versus Li) and increased specific capacity (203 mAh g^−1^ in a lithium half-cell). PI-FTD-NCM811 cathodes also demonstrate high thermal stability, with a heat flow of 253 J g^−1^ at higher temperatures (~230 °C), and have passed flame tests without catching fire. Wang, Y. et al. reported durable PIS binders with ion channels that can be rearranged and copolymerized [41]. The flexible aminopropyl terminated polydimethylsiloxane (PDMS) chains of PIS facilitate fast ion transfer, while the Si-O-Si component of the aromatic polyimide induces complexation reactions with Li+ ions, further enhancing electrochemical performance. NCM811 cathodes using PIS-based binders exhibit a denser and more uniform binder coating layer compared to PVDF-based ones, promoting electron transport and achieving a 94% capacity retention rate after 100 cycles in the voltage range of 2.5–4.3 V. Ni-rich NCMs undergo complex surface chemistry at the CEI due to electrolyte deposition [42]. Moreover, repeated electrolyte circulation distorts the cathode lattice, resulting in poor electrochemical cycling. Polyaniline (PANI) with delocalized conjugated electrons has been shown to effectively improve electron conductivity. PANI is attracting attention as a next-generation binder because the imine nitrogen in PANI can inhibit the dissolution of active materials by coordinating anion groups such as F^−^ in the electrolyte. Li, J. et al. demonstrated that a PANI binder-based Ni-rich layered cathode (LiNi_0.94_Co_0.06_O_2_) significantly improved the capacity retention rate from 47% to 81% of its initial capacity after 1000 cycles and even improved electrode performance at low temperatures (−20 °C).

#### 3.2.3. LiFePO_4_

LiFePO_4_ (LFP) has emerged as a potential cathode material for LIBs owing to its desirable characteristics such as a theoretical capacity of 170 mAh g^−1^, reversibility, low cost, eco-friendliness, and high thermal and chemical stability [43]. However, the limited Li-ion phase-boundary diffusion and low electronic conductivity of LFP prevent the conversion between LiFePO_4_ and FePO_4_, thus restricting its capacity [44]. Poly(3,4-ethylenedioxythiophene):poly(styrenesulfonate) (PEDOT:PSS) is being explored as a polymer material for batteries due to its high electronic conductivity and ambient stability [45]. Del Olmo, R. et al. developed a new binder combining PEDOT:PSS with organic plastic crystals (OIPCs). As a result of the FTIR spectrum of pristine PEDOT:PSS, two different OIPCs (C_2_mpyrFSI and C_2_mpyrTFSI), and each 70/30 P:PSS/OIPC composite, the strongest peaks of the two composites are wider than those of pristine PEDOT:PSS and show different relative intensities. These changes suggest an alteration around the environment of the charged PEDOT and the sulfonate group of the PSS, due to the presence and interaction of OIPC ions. The thermal stability of the materials was determined through thermogravimetric analysis. The decomposition of PEDOT:PSS proceeds with the release of the solvent first below 100 °C, which is generally 20% or more due to the high hygroscopicity of PSS, while the decomposition of C_2_mpyrFSI and C_2_mpyrTFSI starts at 230 °C and 400 °C, respectively. In other words, it is apparent that the thermal stability of the combined binders surpasses that of pristine PEDOT:PSS. These binders improve both electron and ion conductivity, resulting in a capacity retention rate of 99.7% (145.2 mAh g^−1^) for the LFP electrode using a 80/20PEDOT:PSS/N-ethyl-N-methylpyrrolidinium bis(fluorosulfonylimide) (C_2_mpyrFSI) composite binder.

## 4. Separators

### 4.1. Conventional Binders for LIB Separators

A separator serves the purposes of separating the cathode and anode of the battery, preventing electrical short circuits, and at the same time allow rapid transport of ionic charge transport in the electrolyte [46]. Consequently, the separator should act as an excellent insulator while having a structure that enables efficient ion conduction. Polyolefin-based porous films are the most widely commercialized separators to this day. However, as battery safety becomes a vulnerable intrinsic property of polymers, it has become important to increase the thermal stability of polymer separators.

**Figure 5:**
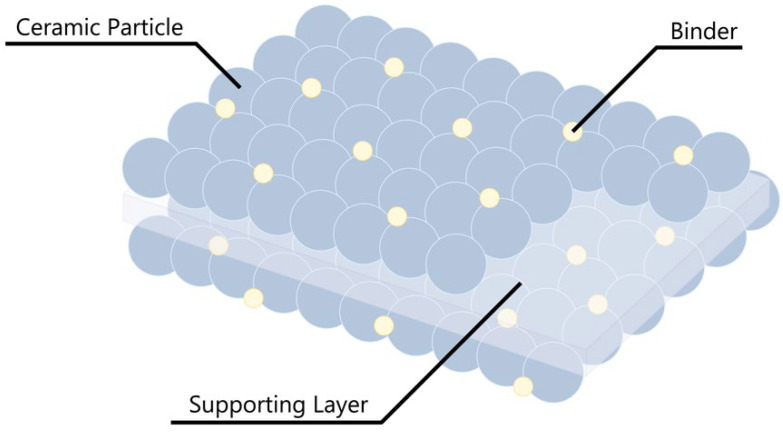
Schematic of the ceramic-coated separator structure.

Takemura, D. et al. developed ceramic powder-based separator (CPS) films to address the limitations of high heat-shrinkage rates in polyolefin-based separators [47]. Figure 5 demonstrates the fundamental CPS structure, and the CPS film developed by Takemura, D. et al. was composed of Al_2_O_3_ powder with different particle sizes and PVDF binders. Thermal analysis revealed that the CPS film did not shrink within any range, whereas the PE film shrank above 90 °C and expanded above 140 °C. In addition, CPS using Al_2_O_3_ powder with a small particle size exhibited excellent circulation characteristics. Thus, the binder holding the inorganic material coated on the polymer membrane is very important in maintaining the performance of the CPS. Subsequently, CPS has been studied under the name of ceramic-coated separator (CCS). LG Chem (now LG Energy Solution), a ceramic-based separator leading company, has further developed Safety Reinforced Separator^®^ (SRS^®^), and SRS^®^ is now a standard for automobile cells.

Since the development of CCS, research has been actively conducted to improve battery performance by improving the thermal properties of the separator using various combinations of inorganic substances and binders that make up the CCS surface coating component. In particular, heat-resistant inorganic substances can effectively suppress the thermal shrinkage of the separator. Jeong, H. S. and Lee, S. Y. introduced a ceramic coating layer consisting of SiO_2_ nanoparticles and a PVDF-HFP binder on both sides of a polyethylene (PE) separator to improve thermal shrinkage and electrochemical performance [48]. Through field emission scanning electron microscope (FE-SEM) photographs, the composite separators have a unique ceramic coating layer consisting of dense SiO_2_ nanoparticles interconnected with a PVDF-HFP binder compared to a pristine PE separator. This structure is similar to the nanoparticle arrangement driven by self-assembly. It was also confirmed that the SiO_2_ coating layer of composite separator showed little change in both the cathode and anode directions even after 200 cycles. The dense SiO_2_ nanoparticles interconnected by a PVDF-HFP binder prevented the separator from experiencing thermal shrinkage, which is similar to an arrangement of nanoparticles driven by self-assembly. Park, J. H. et al. demonstrated a new approach by introducing dense inorganic oxide/polymer binary nanoparticles into the composite layer [49]. They found that using gel polymer electrolytes as a binder in ceramic composite layer-based separators reduced ion conductivity. The close-packed SiO_2_/Poly(methyl methacrylate) binary nanoparticle (hereafter referred to as SiO_2_/PMMA-BNP) coating layers formed a highly porous structure through well-connected interstitial voids, leading to improved discharge capacity and C-rate compared to using a film-type PMMA binder. Zhang, S. et al. developed an alkali CaCO_3_-based composite membrane that neutralizes acidic products to solve the problems caused by HF, which is inevitably present in the LiPF_6_-based electrolytes used currently in the LIBs [50]. The membrane was composed of CaCO_3_ powder and Teflon binder, exhibiting high wettability and excellent capacity.

Generally, polyolefin-based separators have limitations such as low wettability to electrolytes and low thermal stability [51]. Therefore, in order to overcome these problems, studies on new polymers for binder to replace the polyolefin system have been actively conducted. Kim, M. et al. developed a PMMA triple-layer separator based on the idea of excellent thermal stability of inorganic sub-micron particles and their wettability with organic electrolytes [52]. The PMMA membrane with added Al_2_O_3_ powder and PVDF-HFP binder increased its tensile strength more than three times compared to PE and pure PMMA separators. Choi, J. A. et al. achieved high wettability by using a hydrophilic poly(lithium 4-styrene sulfonate) polymer as a binder [53]. They confirmed that thermal deformation was suppressed due to the frame structure of the heat-resistant ceramic powder made of a polymer binder. Moreover, cells using ceramic-coated PE separators containing a high content of poly(lithium 4-styrenesulfonate) (PLSS) showed better capacity retention. Ko, Y. et al. introduced copolyesters (cPET) as a new polymer binder for ceramic composite-coated PE separators [54]. Comparing thermal shrinkage resistance with PVDF binder, they confirmed that the PVDF film contracted 16% at 150 °C and 24% at 200 °C, whereas the cured cPET film showed a shrinkage of 6% at 150 °C and 8% at 200 °C. The thermal properties of cPET-Al_2_O_3_ indicated that the bare PE separator shrank by 70% or more at a temperature of 130 °C or higher, while the cPET-PE separator with a 3 μm composite layer had a shrinkage of only 13% at 170 °C, showcasing great dimensional stability at T_m_ or even higher. The battery cycle test showed a slightly lower capacity retention rate than the bare PE separator, but no undesirable abnormal cycle test results were observed, indicating that the cross-linked cPET binder does not cause harmful reactions that hinder battery operation. Shi, C. et al. introduced a CCS for LIBs using a styrene-butadiene rubber-carboxymethyl cellulose (SBR-CMC) mixed binder, a strong dispersion medium that promotes the uniform distribution of Al_2_O_3_ particles and exhibits high adhesiveness [55]. SBR provides higher flexibility, stronger bonding, and higher heat resistance, while CMC possesses two functional groups (carboxylate anion and hydroxyl group), making it an effective dispersion and thickener agent for aqueous suspensions. Consequently, the SBR-CMC composite binder achieves excellent thermal stability even with a small amount compared to other polymer binders, and as the ceramic coating layer increases, the absorption rate of the liquid electrolyte increases while heat shrinkage decreases.

### 4.2. Next-Generation Separator Binders

Currently, PVDF is widely used as a binder material for LIBs. Despite certain advantages of this binder, there are drawbacks such as the necessity for processing with toxic and volatile solvents like NMP. Both of these materials have high costs due to intricate production and recycling challenges. Organic solvents can pose dangers, hence the necessity to address risk mitigation, safety, and environmental issues by substituting fluorinated binders with fluorine-free aqueous alternatives. Given these factors, water-based adhesives are perceived as the most promising and efficient binders due to their wide accessibility, lower costs, absence of solvent evaporation, safety, and environmental friendliness. Among various water-soluble adhesives, cellulose-derived binders (e.g., CMC) have been garnering increased attention within the realm of advanced LIBs [56]. Enhancing the thermal stability and electrolyte wettability of polyolefin separators can be effectively achieved through surface coating with binders and ceramic particles. However, this approach faces challenges like pore obstruction and a conflict between the adhesion and thermal stability of binders. In their work, Luo, Hui, and their colleagues developed a unique raspberry-like micro-sized polymer (RMP) binder, characterized by a soft core and hard external spheres, utilizing the process of seed swelling polymerization [57]. This microscale RMP binder proves effective in strongly binding the separator and electrode, thereby addressing the pore-blocking issues commonly faced with conventional liner binders. The raspberry-like architecture blends pliability and firmness, thereby ensuring both superior spherical shape-retention capacity and impressive binding strength. The ceramic separator incorporating this RMP binder exhibits reduced thermal contraction and increased ionic conductivity, resulting in improved cycling stability and lowered battery impedance. This research offers fresh insights into the development of innovative binders for ceramic-coated separators, which ensures long-term stability for high-performing LIBs. In summary, researchers have successfully showcased an ideal raspberry-like RMP binder at the microscale level, with excellent high-temperature shape retention and strong binding capabilities. The soft inner core of the binder can absorb electrolytes to secure effective adhesion, while the hard outer particles contribute to overall strength. This RMP binder, compared to traditional liner binders, effectively binds the separator and electrode while preventing pore obstruction. Cells using the RMP binder show a slower rise in impedance during cycling, achieving remarkable capacity and capacity retention. The composite separator with RMP0.15 exhibited a capacity retention rate of 87.5% of its initial capacity after 100 cycles. This research holds the potential to bridge the current knowledge gap in the development of micro-size binders for ceramic-coated separators and presents promising prospects for energy applications demanding long-term stability [57].

Kim, J. Y., and colleagues introduced an innovative process that uses a polyvinyl alcohol (PVA) binder for the in situ surface alteration of hydrophobic separators, leading to enhanced wetting properties with various aqueous slurries [58]. The wettability of the altered separator surface was assessed through dynamic contact angle measurement, revealing a near-zero-degree receding contact angle for the PVA-treated separator. As a proof of concept, aqueous slurries, which typically struggle to coat hydrophobic separators, were uniformly coated using PVA as a surface adjuster. In addition, through the proposed fabrication method, a ceramic-coated separator with enhanced thermal stability and adhesion strength was developed while minimizing the impact on the Li-ion transport. This approach, leveraging cost-effective and environmentally friendly aqueous binders, successfully creates functional separators for diverse advanced batteries with superior characteristics. As demonstrated preliminarily in this work, this method offers limitless potential for application across various battery systems. Additionally, by considering the functional groups of binders, a variety of functional materials with unique surface chemistries can be harnessed using the most appropriate aqueous binders, potentially leading to unforeseen improvements in the battery performance [58].

In an effort to prevent thermal degradation and shrinkage of commercially available polyolefin-based separators in LIBs, Kim, P. S., Le Mong, A., and Kim, D. have introduced a new approach [59]. They used a new binder to coat Al_2_O_3_ particles on the separator. Recognizing the limitations of the commonly used PVDF binder, particularly its weak cohesive binding strength to both the separator and ceramic particles, the researchers synthesized a new semi-interpenetrating polymer network (SIPN)-type binder. This was achieved by cross-linking ethoxylated pentaerythritol tetraacrylate (EPETA) in the presence of PVDF. When employed in a Al_2_O_3_-coated polypropylene/polyethylene/polypropylene (PP/PE/PP) multilayer separator, this SIPN binder demonstrated significantly greater thermal and mechanical stability compared to the original PVDF binder. This is attributed to the enhanced adhesive strength of the polar EPETA component. The polar character of the cross-linked poly(EPETA) also led to an increased wetting and uptake of electrolyte liquid and improved the conductivity of Li+ ion. Consequently, this resulted in the improved electrochemical performance at a high discharge rate [59].

**Figure 6:**
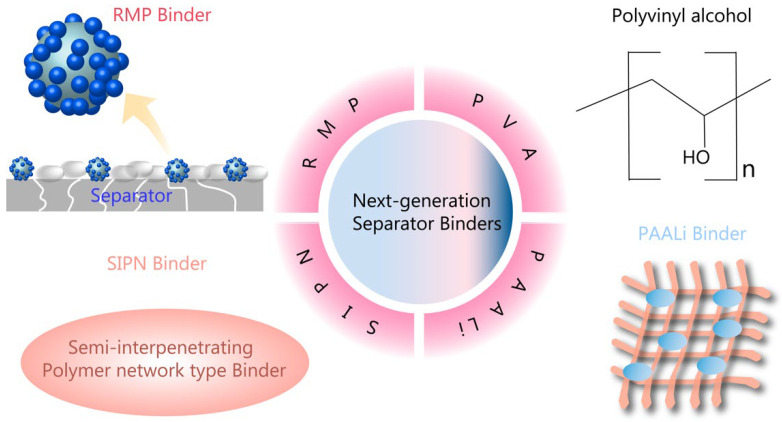
Schematic of next-generation polymer binders for separator.

Li, Menglin et al. created a PI nanofiber membrane. It was created using an electro-spinning technique but was found to have inferior mechanical strength due to fiber slippage, limiting its application in LIBs [60]. They aimed to increase the macro mechanical strength of the PI nanofiber membrane by preventing fiber slippage. Lithium polyacrylate (PAALi), a superior binder, was used to boost the adhesive interaction between the fibers. As a result, the PAALi-treated PI nanofiber membrane displayed a mechanical strength of 16.1 MPa, surpassing the 5.0 MPa of the untouched PI membrane. This binder’s introduction caused the originally loosely packed and unstructured PI nanofibers to cross-link, preventing slippage among the fibers. Interestingly, the PAALi binder did not affect the thermal stability and electrochemical performance of the PI nanofiber separator. Moreover, LiCoO_2_/Li cells equipped with the modified PI nanofiber separators demonstrated improved cycling stability and superior rate performance compared to those using the original PI separators. This indicates that the PAALi binder utilization is a promising strategy to bolster the mechanical strength of nanofiber membranes, facilitating their use in high-power LIBs [60]. Figure 6 introduces four types of next-generation polymer binders for separators. This includes schematic representations of RMP binder and PAALi binder, as well as the chemical structure of polyvinyl alcohol.

## 5. Conclusions and Prospect

In this review, we comprehensively present the conventional and recent developments of polymer binders that comprise LIBs. While most of the research work has been focused on the development of anode and cathode active materials, other components of the battery also have a significant impact on the electrochemical performance of the battery. In particular, the binder plays an important role in stabilizing the microstructure and interface of the electrode and separator. Although PVDF binder has been commonly used in the LIB industry, it has low particle cohesion and electrical conductivity, which are essential for battery performance, and it has expensive and environmentally harmful manufacturing processes. Therefore, in order to develop stable electrodes and separators, it is important to develop more efficient binders.

The volumetric expansion of the anode is known to be a serious factor in the stability and lifespan of LIB. Therefore, it is necessary to apply a binder with better adhesion and elasticity to the anode, and ongoing research is aimed at developing new copolymers and using self-healing polymers to control changes in the volume of the anode. The most important concerns in the cathode are structural stability and capacity. Research has been conducted to suppress the dissolution of an active material and to form a thin and constant CEI layer through a dense and uniform binder-coating layer. Research on a suitable binder to solve the low thermal stability of the polyolefin-based separator is being conducted, and results of increasing electrolyte wettability have also been reported.

The polymer binder occupies a very small part in manufacturing an electrode and a separator, but it plays an important role in electrochemical performance and mechanical and thermal stability. The binder for LIBs must possess favorable electrolyte wettability, strong adhesive properties, and elasticity, which are crucial factors. In addition, instead of using toxic or expensive solvents, eco-friendly and sustainable binder design strategies should be adopted. Since the binder may affect interface reactions such as SEI formation, electrode corrosion, and metal ion dissolution, an in-depth mechanistic study is needed to reveal the basic operating mechanism of the binder.

In the case of next-generation binders, additional research should be conducted because side reactions due to battery aging or severe conditions may have a fatal effect on the battery. In addition, existing binder research tends to rely too much on experimental results because of the lack of theory on binder design, so it is necessary to reduce optimization time by introducing technologies such as machine learning. Moreover, it is important to develop a binder with new functional groups to compensate for the drawbacks of the existing binder. Therefore, future R&D should be conducted in the following directions. First, the criteria for evaluating the mechanical properties (adhesive strength, tensile force, electrolyte uptake, etc.) of the binder must be established. Next, to delve into the impact of the binder on the surface reaction, it is essential to incorporate a theoretical calculation approach. Finally, binders with high electron and ion conductivity should be developed via novel polymers and functional groups. Continuing basic research to optimize polymer binders and understand binder behavior will contribute greatly to the performance improvement of LIB systems in the future.

## Figures and Tables

**Table 1:** Polymer density of PVDF, PAA, and CMC.

Binders	PVDF	PAA	CMC	Ref.
Density g/cm^3^	1.8480 ± 0.0036	1.5121 ± 0.0276	1.6511 ± 0.0148	[18]

**Table 2:** The electrochemical performance of the electrodes (the Si content of 70%) with different polymers as the binder. The current density is C/10, and the specific capacity is the delithiation capacity.

Binders	PVDF	PAA	CMC	Ref.
Specific Capacity (mAh g^−1^, 100th)	118.3	1155.7	1125.5	[18]
Capacity Retention (%, 100th)	8.04	45.17	52.07	[18]

**Table 3:** Comparison of the electrochemical performances for Si electrodes fabricated by PVDF, PAA, and CMC binders.

Binders	Si Content (%)	Electrode Mass Loading (mg/cm^2^)	Electrochemical Performance	Specific Capacity Normalized to Total Electrode (mAh/g)	Ref.
PVDF	80	2.6	~600 mAh/g @ 0.15 A/g, 50th	480	[19]
PAA	80	0.89–1.7	~700 mAh/g @ 0.1 A/g, 50th	560	[20]
CMC	33.3	2.26–2.83	~3000 mAh/g @ ~0.13 A/g, 100th	1000	[21]

**Table 4:** Comparison of the electrochemical performances for various electrodes fabricated using a PVDF binder.

Active Materials	Mixing Ratio	Electrochemical Performance	Capacity Retention (%)	Ref.
Li(Li_0.17_Ni_0.25_Mn_0.58_)O_2_	8:1:1	~238 mAh/g, 100th	80	[28]
Cu-LCO	54:3:10.4	~100.9 mAh/g, 27th	97	[29]
LiNi_0.8_Co_0.15_A_l0.05_O_2_	89.4:8:4.8	~173 mAh/g, 600th	90	[30]

## Data Availability

Not applicable.

## Abbreviations

LIB	Lithium-ion battery
NMP	N-methyl-2-pyrrolidone
EV	Electric vehicle
HEV	Hybrid electric vehicle
PVDF	Polyvinylidene fluoride
SHPET	Self-healing poly(ether-thioureas)
Si	Silicon
LCO	Lithium cobalt oxide
DSL	Dextran sulfate lithium
PI-FTD	Fluorinated polyimide
PIS	Poly(imide-siloxane)
Cyrene	Dihydrolevoglucosenone
NCM	Nickel cobalt manganese oxide
PTFE	Polytetrafluoroethylene
PAN	Polyacrylonitrile
GPE	Gel polymer electrolytes
AMMA	Poly(acrylonitrile-methyl methacrylate)
AN	Acrylonitrile
MMA	Methyl methacrylate
SEI	Solid electrolyte interface
PHT	Poly(3-n-hexylthiophene)
CMC	Carboxymethyl cellulose
PAA	Poly(acrylic acid)
LTO	Lithium titanium oxide
DMF	Dimethylformamide
FTIR	Fourier transform infrared
TGA	Thermogravimetric analysis
CV	Cyclic voltammetry
HPR	Hydroxypropyl polyrotaxane
AB	Acetylene black
HRTEM	High-resolution transmission electron microscopy
S-binder	Si-based binder
Sb-LCO	Lithium cobalt oxide electrode using S-binder
P(VDF-g-VPh)	Vinylphenol-grafted PVDF
CEI	Cathode–electrolyte interface
PANI	Polyaniline
PI	Polyimide
PDMS	Polydimethylsiloxanes
LFP	Lithium iron phosphate
PEDOT:PSS	Poly(3,4-ethylenedioxythiophene):poly(styrenesulfonate)
OIPC	Organic plastic crystal
C_2_mpyrFSI	N-ethyl-N-methylpyrrolidinium bis(fluorosulfonylimide)
CPS	Ceramic powder-based separator
CCS	Ceramic-coated separator
SRS^®^	Safety Reinforced Separator^®^
FE-SEM	Field emission scanning electron microscope
SiO_2_/PMMA-BNP	SiO_2_/Poly(methyl methacrylate) binary nanoparticle
PLSS	Poly(lithium 4-styrenesulfonate)
cPET	Copolyester
SBR	Styrene-butadiene rubber
RMP	Raspberry-like micro-sized polymer
PVA	Polyvinyl alcohol
SIPN	Semi-interpenetrating polymer network
EPETA	Ethoxylated pentaerythritol tetraacrylate
PP	Polypropylene
PE	Polyethylene
PAALi	Lithium polyacrylate
